# Supplementary material for: A Novel Enterococcus-Based Nanofertilizer Promotes Seedling Growth and Vigor in Wheat (Triticum aestivum L.)
Source: Plants (Basel). 2024 Oct 14;13(20):2875. doi: 10.3390/plants13202875 (PMC11510893; doi:10.3390/plants13202875)
Supplement: Supplementary file 1 [file plants-13-02875-s001.zip › plants-3172191-supplementary.pdf]

Supplementary Materials

A Novel *Enterococcus*-based Nanofertilizer Promotes Seedling Growth and Vigor

Salma Batool[1], Maryam Safdar[1], Saira Naseem[2], Abdul Sami[3], Rahman Shah Zaib Saleem[4], Estibaliz Larrainzar[5], Izzah Shahid[5*]

[1]Department of Basic and Applied Chemistry, Faculty of Science and Technology, University of Central Punjab, 1- Khayaban –e- Jinnah Road, Johar Town, Lahore, 54782, Pakistan
[2]Department of Biotechnology, Faculty of Science and Technology, University of Central Punjab, 1- Khayaban –e- Jinnah Road, Johar Town, Lahore, 54782, Pakistan
[3]H.A. Shah & Sons Group of Companies, Islamabad, 46000, Pakistan
[4]Department of Chemistry and Chemical Engineering, SBA School of Science and Engineering (SBASSE), Lahore University of Management Sciences (LUMS), Lahore, 54792, Pakistan
[5]Institute of Multidisciplinary Research in Applied Biology, Public University of Navarre, Campus Arrosadia, Pamplona 31006, Spain

[*]Corresponding Author: Izzah Shahid
izzah.shahid@unavarra.es

Table S1. Biochemical characterization of spinach isolates using QTS-24 identification kits and Bergey's Manual for identification.

Sr. No	1	2	3	4	5	6	7	8	9	10	11	12	13	14	15	16	17	18	19	20	21	22	23	24	25	Identifications by QTS-24 kit
Bacterial Isolates	ONPG	CIT	MALO	LDC	AHD	ODC	H_2S	UREA	TDA	IND	VP	GEL	GLU	NO_2/NO_3	MALT	SUC	MANN	ARAB	RAHM	SORB	INOS	ADO	MEL	RAF	CO	
SR1	+	-	+	+	+	+	-	-	+	+	-	+	+	+/+	+	+	+	+	+	+	-	-	+	+	+	*Vibrio* sp.
SR2	+	+	+	-	+	-	-	-	+	+	+	+	+	+/+	+	+	+	+	+	+	-	-	+	+	+	*Aeromonas hydrophilic*
SR3	+	+	+	-	+	+	-	-	+	+	+	+	+	+/+	+	+	+	+	+	+	-	-	+	+	-	*Burkholderia pseudomallei*
SR4	-	+	+	+	+	+	-	-	+	-	+	+	+	+/+	+	+	+	+	+	-	-	-	+	+	-	*Vibrio* sp.
SR5	+	-	+	-	+	-	-	-	+	+	+	-	+	+/+	+	-	+	+	+	+	-	-	+	+	+	*Aeromonas hydrophila*
SR6	+	+	+	+	+	+	-	-	+	+	+	+	+	+/+	+	+	+	+	-	+	-	-	+	+	+	*E. coli*
SR7	-	-	+	+	+	-	-	-	-	+	-	+	+	-/+	+	+	+	+	+	+	-	-	+	+	+	*E. coli*
SR8	+	+	+	-	+	-	-	-	+	-	+	-	+	+/+	+	+	+	+	+	-	-	-	+	+	+	*Burkholderia pseudomallei*
SR9	+	+	+	-	+	-	-	-	+	+	+	+	+	+/+	+	-	-	+	+	-	-	-	+	+	-	*Pesteurella pneumotrppica*
SR10	+	+	+	-	+	+	-	-	+	+	+	+	+	+/+	+	-	+	+	-	+	-	-	+	+	+	*Vibrio halophilic*
SR11	+	+	+	-	+	-	-	-	+	-	+	+	+	+/+	+	-	-	+	+	+	-	-	+	+	-	*Aeromonas hydrophilla*
SR12	+	+	+	-	-	-	-	-	+	+	+	+	+	+/+	+	-	+	+	+	+	-	-	+	+	+	*Aeromonas hydrophilla*

(+): Positive, Negative: (-)

Symbols mentioned in table S1: ortho-Nitrophenyl-β-ga1actosidase (ONPG), sodium citrate (CIT), malonate (MALO), lysine decarboxylase (LDC), arginine dihydrolase (ADH), ornithine decarboxylase (ODC), hydrogen sulfide (H2S), urease (UREA), tryptophan deaminase (TDA), indole (IND), Voges-Proskauer (VP), gelatin hydrolysis (GEL), glucose (GLU), nitrate reduction, nitrogen production (NO_3/N_2), maltose (MALT), sucrose (SUC), mannitol (MANN), arabinose (ARAB), rhamnose (RHAM), sorbitol (SORB), inositol (INOS), adonitol (ADO), melibiose (MEL), raffinose (RAF) and cytochrome oxidase (CO).

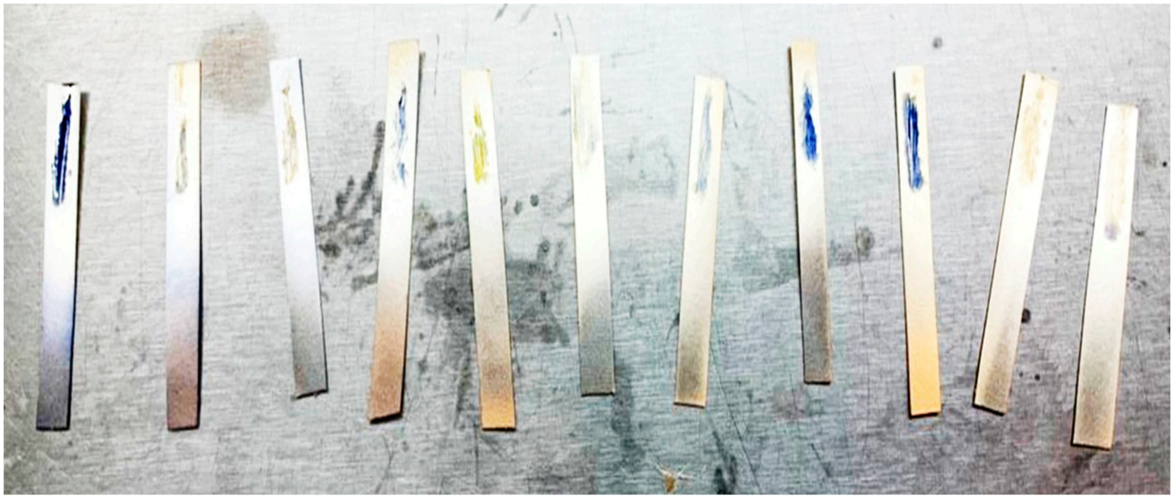

Figure S1. Bacterial isolates indicating the CO production

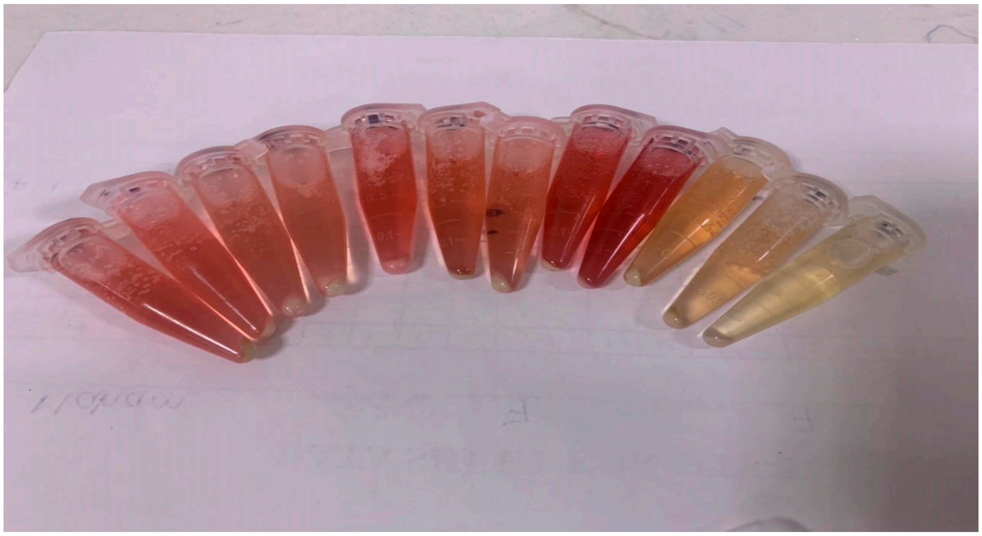

Figure S2. Bacterial isolates showing the indole acetic acid production.

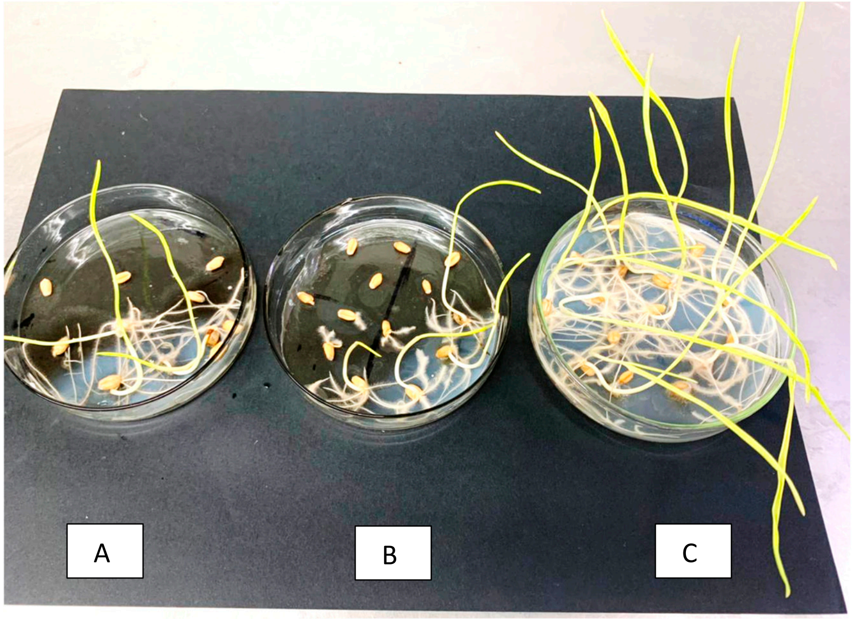

Figure S3. Effect of PGPR bacterial isolates on wheat growth promotion, (A) control, (B) *Aermonas hydrophilla*, (C) *Enterococcus* sp.

Table S2. Table of vigor index and germination rate of wheat seedlings.

Sr. No	Treatments	Vigor Index	Germination (%)
1	Control	680± 0.03	65
2	SR12 (non PGPR)	804± 0.01	70
3	SR1	1080± 0.04	80
4	SR2	1370± 0.01	85
5	SR3	1865± 0.08	96
6	SR9	1928± 0.04	98
7	SR10	1423± 0.09	90

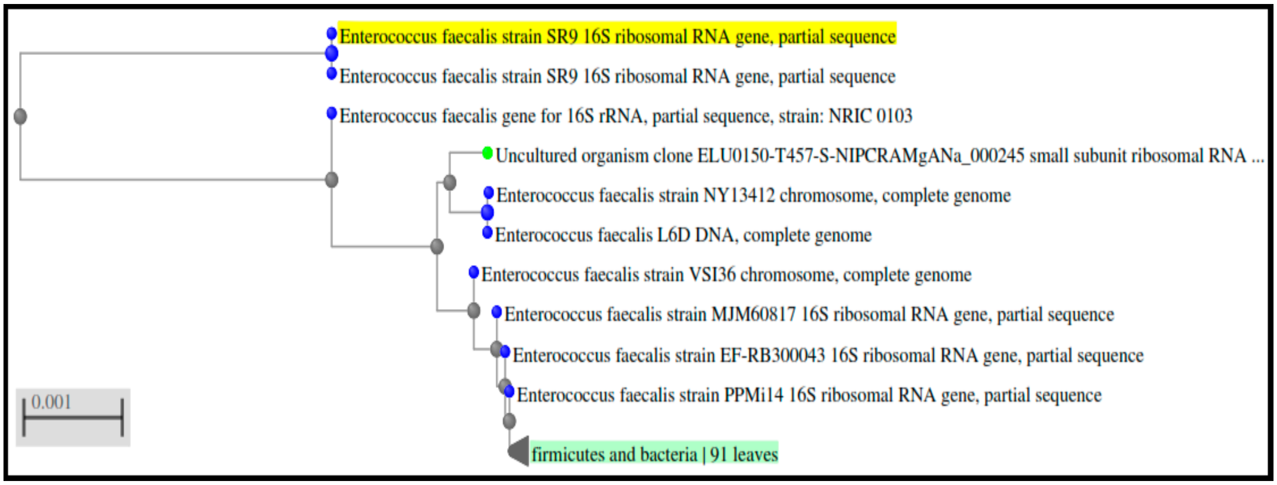

Figure S4. Phylogenetic tree of SR9 constructed using neighbor-joining method. SR9 showed close homologies with *Enterococcus* sp. strains. The phylogenetic tree was constructed NCBI BLAST tool and the evolutionary history was inferred using Neighbor-Joining method. Evolutionary distances were computed using the Maximum Composite Likelihood Method and are in the units of the number of base substitutions per site. The analysis involved fourteen nucleotide sequences. All positions containing gaps and missing data were eliminated. There were total of 1137 positions in the final dataset. The blue nodes indicate Firmicutes used in analysis, the green node shows uncultured bacterium, yellow highlight at the top is the query sequence and the green highlight at the bottom is from type material used as reference.

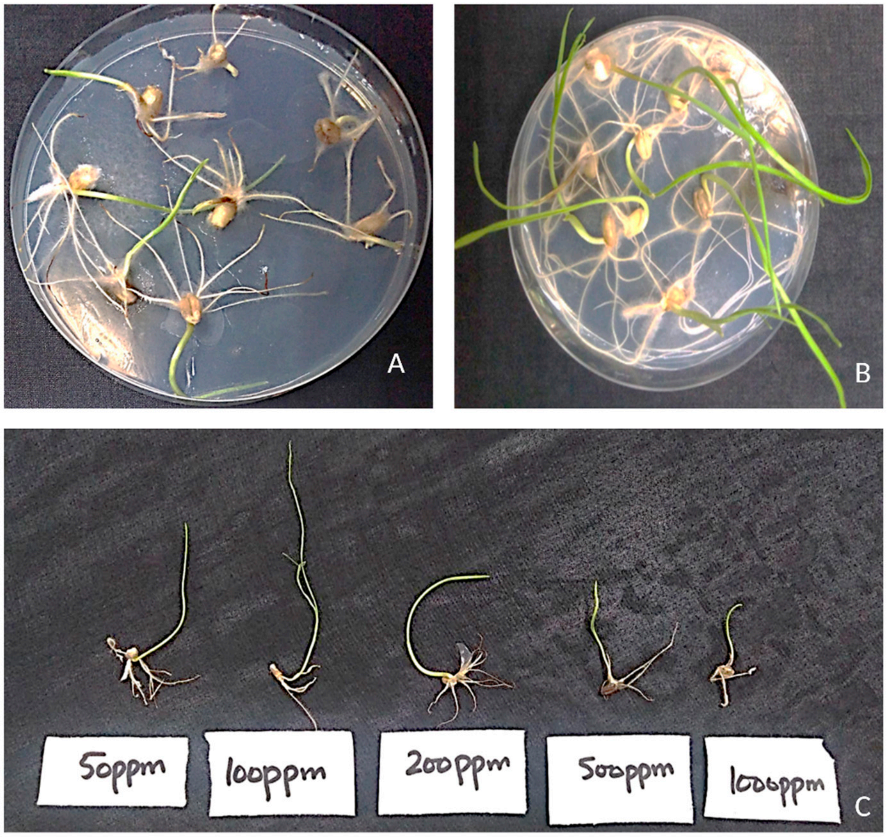

Figure S5. Phytotoxicity assessment of SR9AgNPs on wheat seedlings. Wheat seedlings treated with 1000 ppm of SR9AgNPs (A), and 100 ppm of SR9AgNPs (B). Variation in growth of wheat seedlings following treatment with different concentrations of SR9AgNPs (C) and assessment of vigor indices and percentage germination in response to treatment.

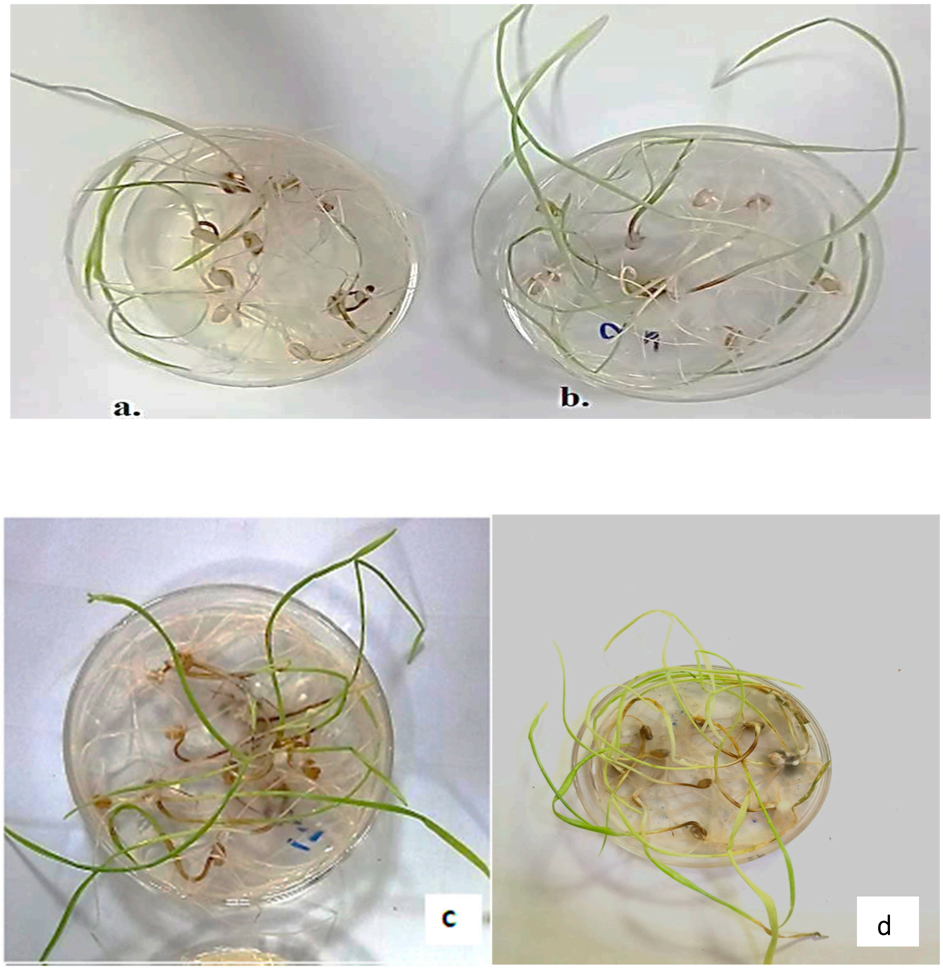

Figure S6. Wheat seeds germination. a) non-treated seeds, b) treatment with SR9, c) treatment with SR9AgNPs and d) seeds treated with SR9 and AgNPs.

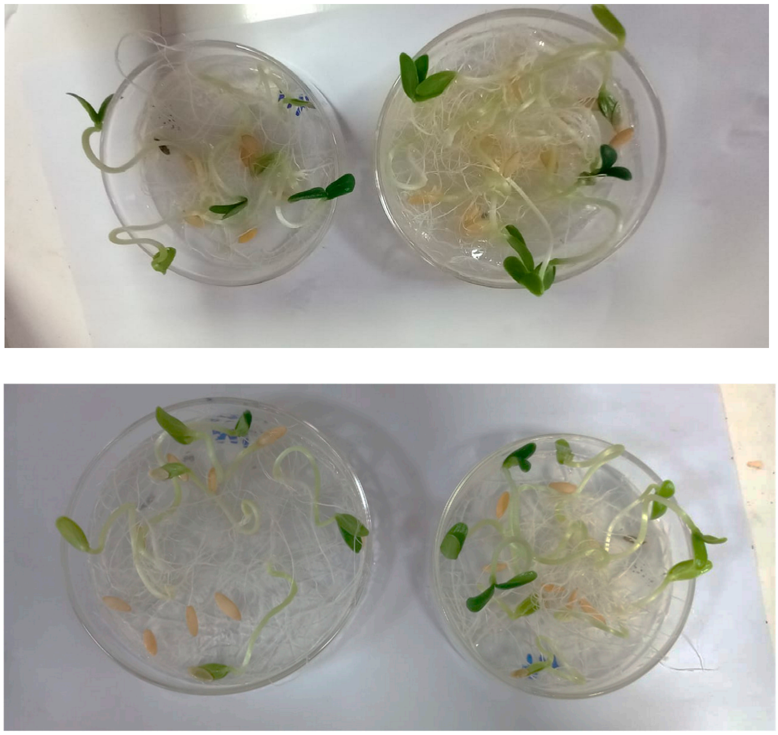

Figure S7. Germination of cucumber seeds in a) control b) treatment with SR9 isolate (biofertilizer) c) AgNPs treatment and d) combination with SR9 isolate and SR9AgNPs.

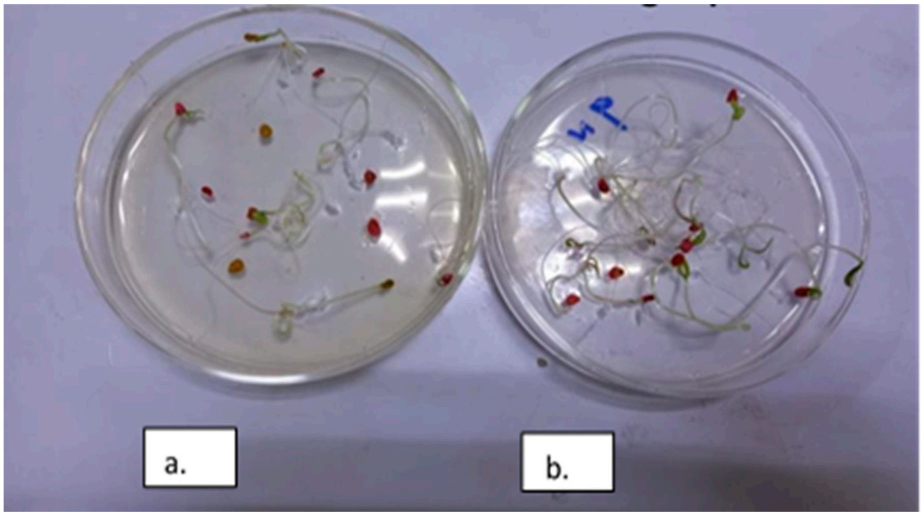

Figure S8. Demonstration of germination of tomato seeds with a) control b) AgNPs treatment group.
